# Nasopharyngeal leiomyomatous hamartoma: case report

**DOI:** 10.1186/1472-6815-14-5

**Published:** 2014-04-29

**Authors:** Takanori Nishiyama, Yasumasa Kato, Yuh Baba

**Affiliations:** 1 Department of Otolaryngology, Nasu Red Cross Hospital, 1081-4 Nakatahara, 324-8686 Ohtawara City, Tochigi, Japan; 2 Department of Oral Function and Molecular Biology, Ohu University, 31–1 Misumido Tomita-machi 963-8611 Koriyama City, Fukushima, Japan; 3 Department of General Clinical Medicine, Ohu University, 31-1 Misumido Tomita-machi, 963-8611 Koriyama City, Fukushima, Japan

**Keywords:** Hamartoma, Nasopharynx, Otolaryngologic tumor

## Abstract

**Background:**

Fibroma, neurofibroma, and papilloma are the most commonly encountered benign lesions in the nasopharynx. Hamartomas are non-neoplastic overgrowth of mature/differentiated tissue indigenous to the specific part of the body in which they develop. Most hamartomas are located in the liver, spleen, lungs, and pancreas. However, nasopharyngeal hamartoma is rare.

**Case presentation:**

We describe here a 77-year-old Japanese woman who presented with a mass arising from the left lateral wall of the nasopharynx. Computed tomography (CT) revealed a soft tissue mass without bony erosion, suggesting that the mass was a benign tumor such as a fibroma. Pathological examination showed that the mass was a leiomyomatous hamartoma.

**Conclusion:**

To our knowledge, this is the first report of a leiomyomatous hamartoma in the nasopharynx. Although leiomyomatous hamartoma in the nasopharynx is extremely rare, it should be kept in mind during differential diagnosis.

## Background

Hamartomas are non-neoplastic overgrowth of mature/differentiated tissue indigenous to the specific part of the body in which they develop [1]. Most hamartomas are located in the liver [2], spleen [3], lungs [4], and pancreas [5], although several hamartomas in the nasopharynx have been reported [1,6,7]. Hamartomas are classified as epithelial, mesenchymal, or mixed epithelial and mesenchymal types. Respiratory epithelial adenomatoid hamartoma, a subtype of epithelial hamartoma, is the most common type of hamartoma in the nasal cavity or nasopharynx; the clinicopathological features of 31 such patients have been described [8]. Mesenchymal hamartomas are much rarer than epithelial type hamartomas. Mesenchymal hamartomas can be subclassified as chondroid, chondromesenchymal, angiomatous, lipomatous, and leiomyomatous hamartomas, depending on their major tissue type [7]. We describe here a patient with a nasopharyngeal leiomyomatous hamartoma.

## Case presentation

**Figure 1:**
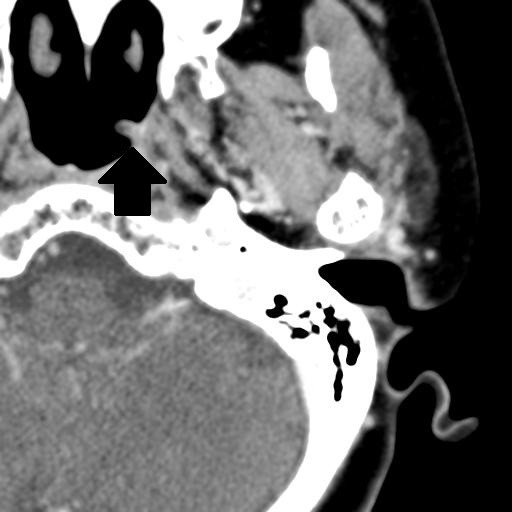
Axial CT images, revealing a small mass almost 5 mm in diameter at the left wall of the nasopharynx.

**Figure 2:**
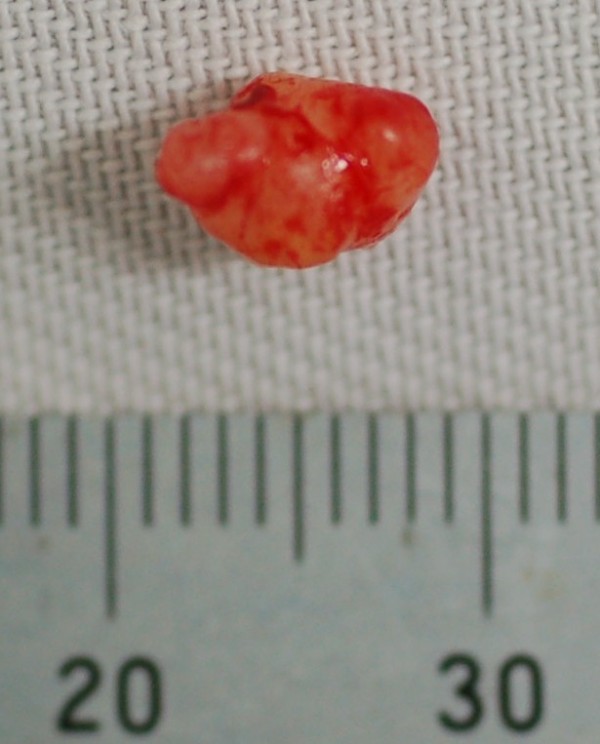
Gross examination of the excised specimen, showing an oval lesion with smooth surface.

**Figure 3:**
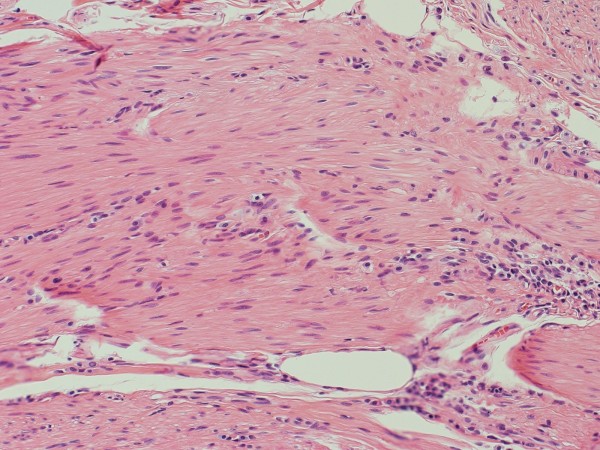
Pathological features of the specimen, showing interlacing bundles of smooth muscles (Hematoxylin and Eosin; 400x).

**Figure 4:**
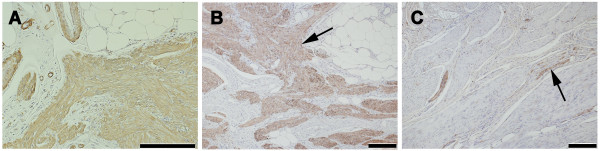
**The histopathological study of leiomyomatous hamartoma in immune-histochemical stain (bar; 100 μm). A:** Positive reaction with smooth muscle actin in the muscle bundles. **B:** Positive reaction with desmin in the muscle bundles (arrow), but negative in the nerve. **C:** Positive reaction with S-100 protein in the nerve (arrow), but negative in smooth muscle bundles.

A 77-year-old Japanese woman with no significant previous medical history presented at our hospital for a cheek swelling sensation during the previous week. Her general condition was very good, without fever or pain, and her cheek was not swollen. Nasal fiberscopic analysis revealed a small mass at the left wall of the nasopharynx. Its smooth surface suggested a benign lesion. Computed tomography (CT) without contrast enhancement showed a mass almost 5 mm in diameter at the left wall of the nasopharynx (Figure 1). The mass was excised by trans-nasal endoscopic surgery under general anesthesia (Figure 2). Pathologic examination showed cells with the mature phenotype of smooth muscle, but arranged haphazardly (Figure 3). Immunohistochemical labeling was performed using an antibody against smooth muscle actin (DAKO, Carpinteria, CA; dilution 1:50), S-100 protein (DAKO, dilution 1:200) and desmin (DAKO, dilution 1:200). Immunoreaction were clearly positive in smooth muscle cells fascicles (Figure 4A and B) (positive immunoreactions for smooth muscle actin and desmin) and nervous tissue (Figure 4C) (positive immunoreaction for S-100). Finally, the patient was diagnosed with a nasopharyngeal leiomyomatous hamartoma. To date, almost 2 years after surgery, there has been no evidence of recurrence, and her mucosa remains healthy.

## Conclusions

Hamartomas are non-neoplastic overgrowth of mature/differentiated tissue indigenous to the specific part of the body in which they develop [1]. Most hamartomas are located in the liver [2], spleen [3], lungs [4], and pancreas [5], but some have been reported in the nasopharynx [1,6,7]. Hamartomas are classified as epithelial, mesenchymal, and mixed epithelial and mesenchymal types. Respiratory epithelial adenomatoid hamartomas are the most common subtype in the nasal cavity or nasopharynx [8]. Mesenchymal hamartomas, which are much less common than epithelial hamartomas, can be subdivided into chondroid, chondromesenchymal, angiomatous, lipomatous, and leiomyomatous hamartomas, depending on the preponderant tissue [7]. Although several leiomyomatous hamartomas have been observed in the oral cavity [9,10], none to date has been reported in the nasopharynx. To our knowledge, this may be the first report of a nasopharyngeal leiomyomatous hamartoma.

Immunohistochemical characteristics are essential in differentiating leiomyoma. The smooth muscle actin and S-100 protein are the most commonly used markers in confirming the histomorphologic findings in leiomyomatous hamartoma [10]. However, use of desmin has been reported [11], because expression of smooth muscle actin is not specific for smooth muscle cells, but is also expressed by myofibroblasts. The S-100 protein is normally present in cells derived from the neural crest, chondrocytes, adipocytes, myoepithelial cells, macrophages, and keratinocytes. Adipose tissue and nerve fibers may also have positive staining but smooth muscle have negative staining. So, S-100 protein can be a key immunohistochemical marker to differentiate leiomyomatous hamartoma from leiomyoma.

The pathogenesis of nasopharyngeal leiomyomatous hamartoma is not clear. Leiomyomatous hamartomas in the oral cavity tend to occur in the midline of the maxillary gingiva and tongue [9,10]. This may have been due to an embryogenic error occurring during the fusion of the primary palate with the lateral palatine processes during the formation of the palate [11]. The mass in our patient was located in the left lateral wall of the nasopharynx, suggesting that it may have arisen from smooth-muscle cells in the left wall of the nasopharynx, caused by unknown origin, rather than through embryogenic error. In summary, we have described a patient with a nasopharyngeal leiomyomatous hamartoma. To our knowledge, this may be the first report of a leiomyomatous hamartoma in the nasopharynx.

## Consent

Written informed consent was obtained from the patient for publication of this Case report and any accompanying images.

## Abbreviation

CT: Computed tomography.

## Competing interests

The authors declare that they have no competing interests.

## Authors’ contributions

The author YB has made the study design and concept, drafted the manuscript, and made critical review. TN has obtained the data and figures, and drafted the manuscript and references. YK has made a critical review for the manuscript and added comments to discussion. All authors read and approved the final manuscript.

## Pre-publication history

The pre-publication history for this paper can be accessed here:

http://www.biomedcentral.com/1472-6815/14/5/prepub
